# Supplementary material for: PICTUREE—Aedes: A Web Application for Dengue Data Visualization and Case Prediction
Source: Pathogens. 2023 May 29;12(6):771. doi: 10.3390/pathogens12060771 (PMC10301560; doi:10.3390/pathogens12060771)

Predicting Insect Contact and Transmission Using histoRical Entomological and Environmental data (PICTUREE)

PICTUREE—Aedes (Dengue)

User Guide

Tanvir Ferdousi, Chunlin Yi

Network Science and Engineering Group (NetSE)

Kansas State University

Table of Contents

Table of Contents 2

Introduction 1

Accessing the PICTUREE--Aedes 1

The graphical interface 1

Selection methods 3

- Point selection 3
- Circle selection 3
- Ecoregion selection 4
- Entire map selection 5

Displaying data and simulation results 5

- Weather stations 5
- Occurrence data (dengue & vector) 6
- Google trends 6
- Weather observations 7
- Reanalyzed data 8
- Vector map 9
- Dengue outbreak case prediction 10
- Dengue epidemic potential 10

Data Sources

Item	Source	Remarks
Daily weather observation data from Global Surface Summary of the Day – GSOD	NCEI- NOAA	Updated weekly
Global occurrence data on *Aedes aegypti* and *Aedes albopictus*	Kraemer, Moritz U. G. et al. (2017), Data from: The global compendium of Aedes aegypti and Ae. albopictus occurrence, v2, Dryad, Dataset, https://doi.org/10.5061/dryad.47v3c	Time range 1960 - 2014
Global occurrence data on dengue	Kraemer, Moritz U. G. et al. (2017), Data from: The global compendium of Aedes aegypti and Ae. albopictus occurrence, v2, Dryad, Dataset, https://doi.org/10.5061/dryad.47v3c	Time range 1960 – 2012
Monthly *Aedes aegypti* and *Aedes albopictus* suitability maps	Walter Reed Biosystematics Unit (WRBU)	Monthly Estimates for 2 mosquito vectors
Country-wise Google trends data on keywords 'dengue' and 'fever'	Google Trends	2015 – 2020
Reanalysis environmental data	ERA5 hourly data on single levels from 1979 to present	2018 - 2020
TNC terrestrial ecoregions	-Olson, D. M. and E. Dinerstein. 2002. The Global 200: Priority ecoregions for global conservation. (PDF file) Annals of the Missouri Botanical Garden 89:125-126.<br>-The Nature Conservancy, USDA Forest Service and U.S. Geological Survey, based on Bailey, Robert G. 1995. Description of the ecoregions of the United States (2nd ed.). Misc. Pub. No. 1391, Map scale 1:7,500,000. USDA Forest Service. 108pp.<br>-The Nature Conservancy (2003), based on Wiken, E.B. (compiler). 1986. Terrestrial ecozones of Canada. Ecological Land Classification Series No. 19. Environment Canada, Hull, Que. 26 pp. + map.	

Introduction

The PICTUREE--Aedes is designed to enable convenient display of spatial and temporal data. These data may include raw or processed environmental measurements, simulation results, and predicted incidence. The backend of the tool consists of a NodeJS server running with PostgreSQL. The frontend is written in JavaScript which uses several open source plugins to display the applications. The PICTUREE--Aedes can be viewed in a standard web browser by using the URL of the map server.

Accessing the PICTUREE--Aedes

The PICTUREE--Aedes is not open to public access. At the time of writing this document, this can be accessed only from inside the Kansas State University network. Outside clients may use the K-State VPN to connect to the network first and then access it. The PICTUREE--Aedes can be accessed using the following URL,

http://10.7.168.148:3254/map

The above URL may change in the future (if the server is relocated, or if there is a change in network configuration). It is recommended to use Mozilla Firefox or Google Chrome to access the application as some browsers may not support or display some features properly. Pop-ups must be allowed to use some data plot functionalities which open new browser windows as popups.

The instructions on how to setup the K-State VPN can be found here,

https://www.k-state.edu/its/security/secure-data/vpn/

The graphical interface

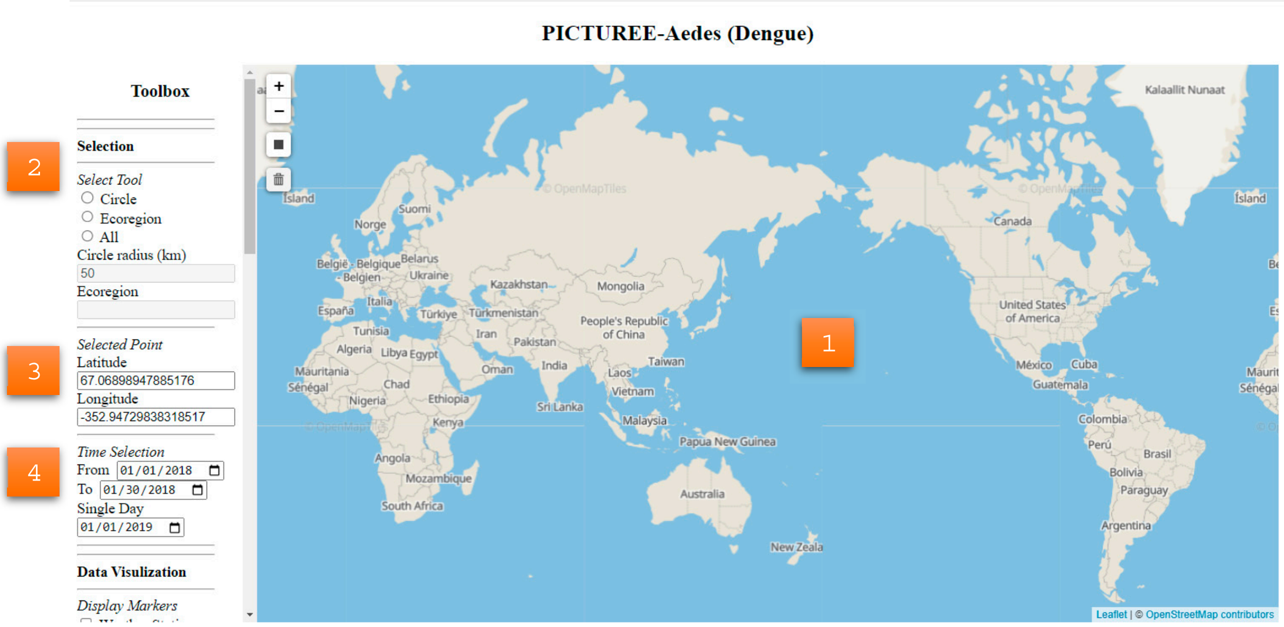

Figure 1: The main screen of the PICTUREE--Aedes.

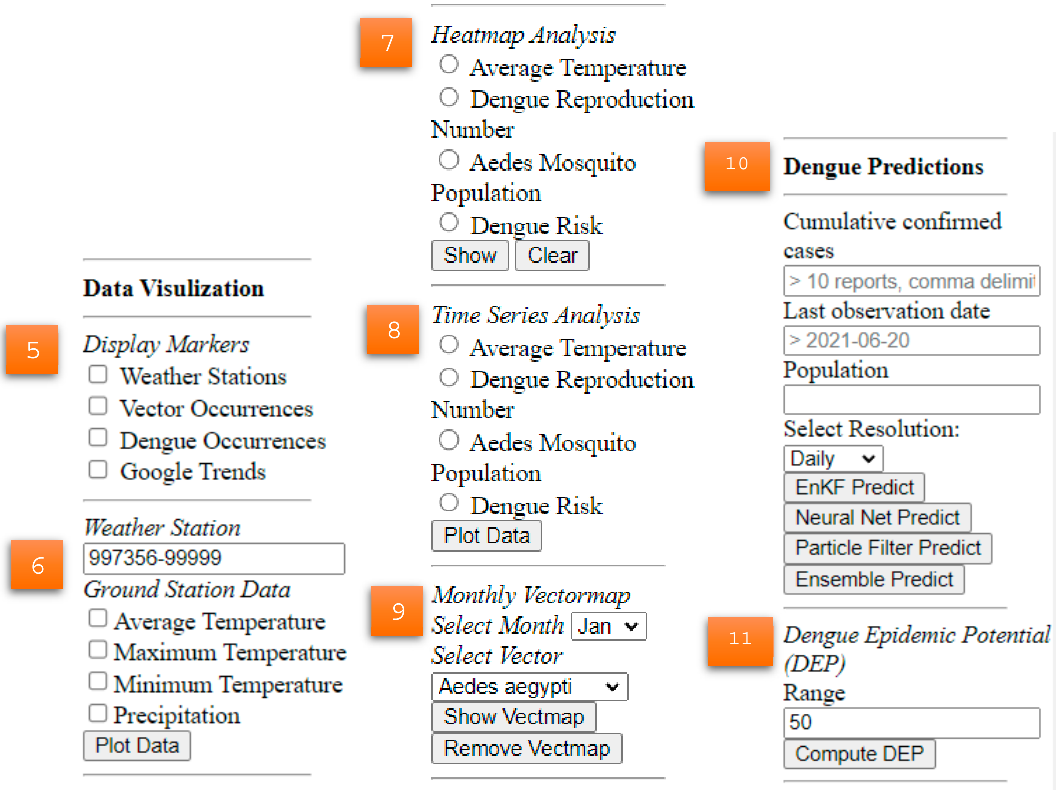

Figure 2: The remaining items of the Toolbox (scrolled down)

Once the browser has loaded the PICTUREE--Aedes, it looks like the images above (Fig. 1 and 2). Different sections of the user interface are marked with orange boxes with numbers for the sake of this description.

The main part of the interface is the map window in the middle (1). It shows a 2D projection map of the entire earth. The map window supports panning and zooming. It can be panned by clicking and dragging the mouse cursor or using the arrow keys in the keyboard when it is in focus. The mouse wheel can be used to zoom in or out of the map. There are also buttons (+ and -) on the top-left corner of the map window which can be used to change zoom levels.

On the left side of the user interface, there are different tools to aid with data selection and visualization (marked with numbers 2 to 11). The selection tools (2) lie on top. There are different ways to select areas in the map: circle, ecoregion, or all (entire map). If the circle tool is used, a radius can be specified to set the size of the circle. If the ecoregion tool is used and an area is selected, the name of the ecoregion will appear in the box marked by Ecoregion. The area marked by (3) displays the co-ordinates when a point is selected. A point can be selected anytime by clicking on the map with the mouse pointer. The time selection boxes (4) can be used to select a time range for temporal data (occurrences, trends, ground station data etc.) visualization or a specific day for spatial data visualization.

The next part (5) of the UI enables several spatial visualization markers. The locations of weather stations, vector and dengue occurrences can be selected using different selection tools from (2). Google search trends data are not available at arbitrary spatial resolutions, rather it is available country-wise. Hence, the selection tools do not apply to it. Weather observation data are collected from ground stations. Once weather station markers are displayed on the map, they can be selected, and the collected data can

be observed for specified time ranges. The UI area marked by (6) shows the selected station id and gives options to select observation variables (temperature, precipitation etc.). After the selection of station, time range, and observation variables, the **Plot Data** button can be used to display temporal observation data in graphical plots.

The tool also supports visualization of spatial data (in the form of heatmaps) which can be selected from the toolbox marked by (7). There is a tool on the upper left corner of the map window (1) which can be used to draw rectangles. These rectangles mark areas for spatial visualization of available data (gridded reanalysis data on temperature or mosquito vector prediction results). The UI area (8) helps with temporal visualization of such data (reanalysis temperature/ vector prediction). The UI area marked by (9) shows some monthly computed global maps of vector suitability.

The UI area marked by (10) is the prediction tool which is able to predict the dengue incidence in the next three weeks if the users input at least 10 cumulative case count, last observation date, the location, and the population of the outbreak region. Four predictive tools can be selected which are ensemble Kalman filter prediction, neural network prediction, particle filter prediction, and ensemble prediction. The tool can compute the dengue epidemic potential (DEP) for any location on the map using the tools marked by (11). By clicking and selecting a point on the map and specifying a range to look for weather stations, the DEP can be computed using the simulator running in the backend server.

Selection methods

Point selection

Point selection is always enabled and occurs by default. Clicking on any point in the map window (1) will extract the coordinate of that exact point and show it in the boxes in (3).

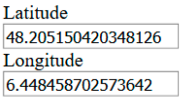

If the elevation tool is running in the server, the PICTUREE--Aedes will also collect the elevation for that point and show as popup.

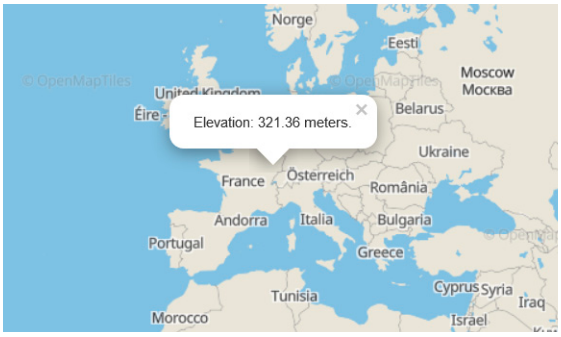

Circle selection

To select using circles, first the Circle tool must be selected. Once selected, the circle radius (km) can also be specified which defaults to 50 km.

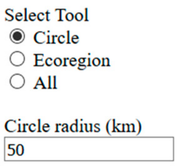

Now, if we click on the map window, it will draw a circle around that point of click with the specified radius.

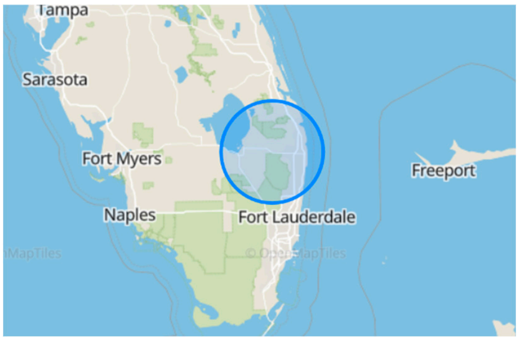

Clicking on another point will simply remove the previously drawn circle and redraw another at the new point.

Ecoregion selection

To select entire ecoregions, first the Ecoregion tool must be selected.

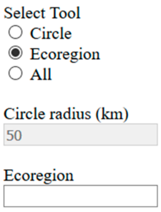

Now, if we click on a point in the map window, it will find out the ecoregion the point lies on and draw it in the map. The Ecoregion textbox will show the name of the selected ecoregion.

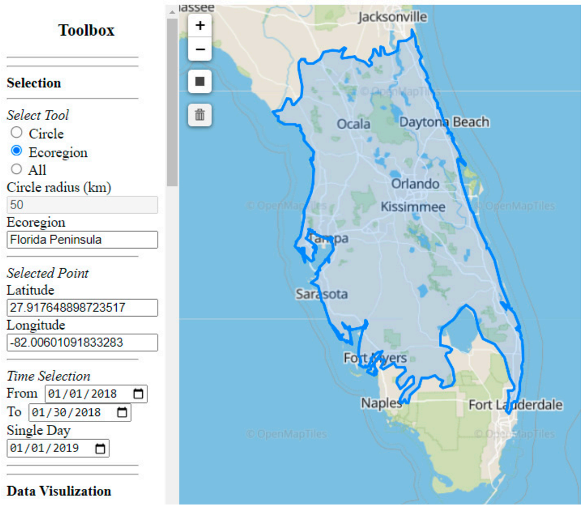

Clicking on another point will simply remove the previously selected region and find the ecoregion for the new point.

Entire map selection

Selecting All in the selection tool will enable global spatial data display. If some markers are selected, those will be displayed for the entire map.

Displaying data and simulation results

Weather stations

To see where the ground weather stations are located, first we must choose a selection tool: **Circle**, **Ecoregion**, or **All** from (2). Next, we must select the **Weather Stations** checkbox under **Display Markers** in (5). Now, if we click on the map, it will show the locations of the weather stations based on the select tool.

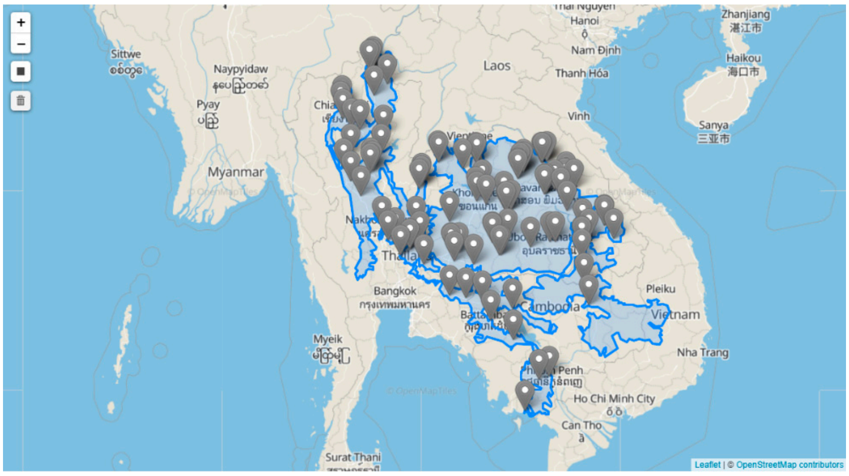

The stations are shown in grey markers. If we click on a marker, a popup will show with the station name as hyperlink. If we click on that link, the station will be selected. The id of the selected station will be displayed in the Station Id box.

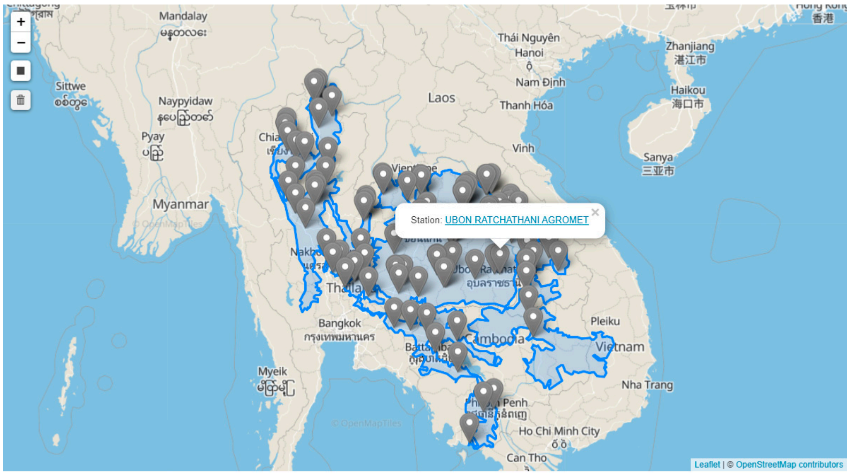

This is the essential step that should be done to select a station before we can display temporal weather observation data (discussed later in this manual).

Occurrence data (dengue & vector)

To see where the occurrences of dengue or its vectors have been recorded, first we must choose a selection tool: Circle, Ecoregion, or All from (2). To view dengue occurrence records, we need to select the **Dengue Occurrences** checkbox under **Display Markers** in (5). Now, if we click on the map, it will show the locations where dengue has occurred before based on the select tool.

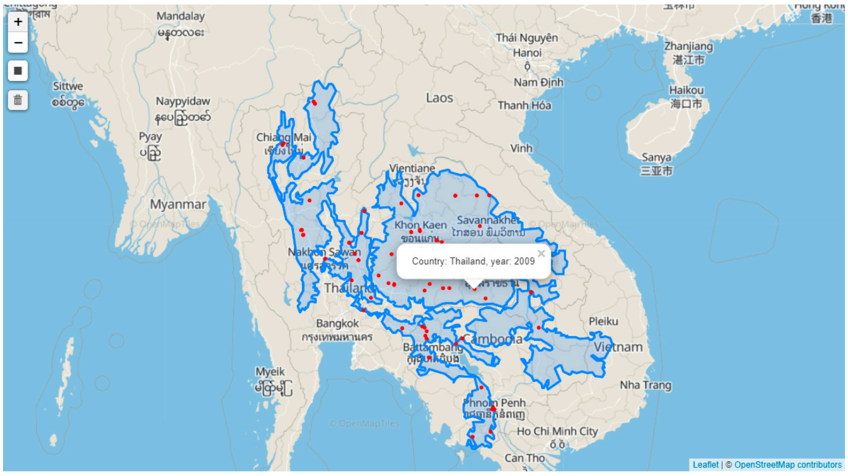

The occurrence records are shown in red dots. If we click on a dot, a popup will show with the country name and the year.

To view vector occurrence records, we need to select the **Vector Occurrences** checkbox under **Display Markers** in (5). Now, if we click on the map, it will show the locations where the occurrence of *Aedes aegypti* and *Aedes albopictus* vectors have been recorded depending on the select tool.

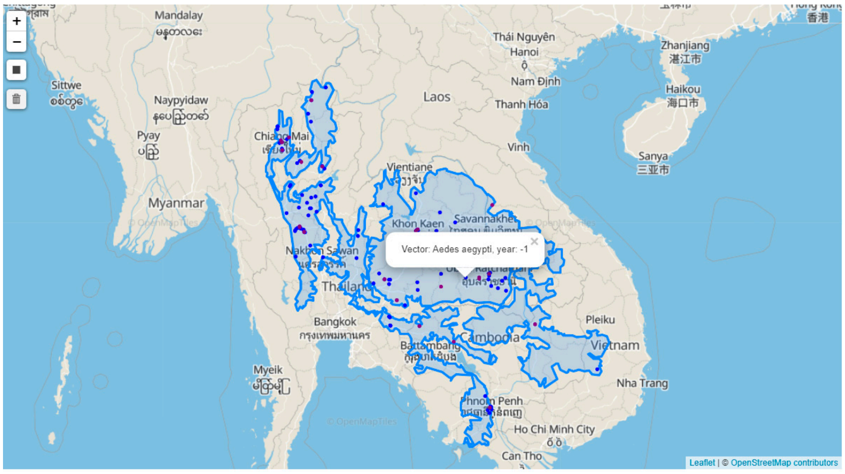

The blue dots correspond to *Aedes aegypti* occurrences and the purple dots correspond to *Aedes albopictus* occurrences.

Google trends

Information on Google search trends are available on a country basis. To view such trends, we do not need the Select Tool. Simply clicking on any point on the map will bring up blue markers on nearby

countries to view country specific temporal data when **Google Trends** checkbox under **Display Markers** (5) is marked.

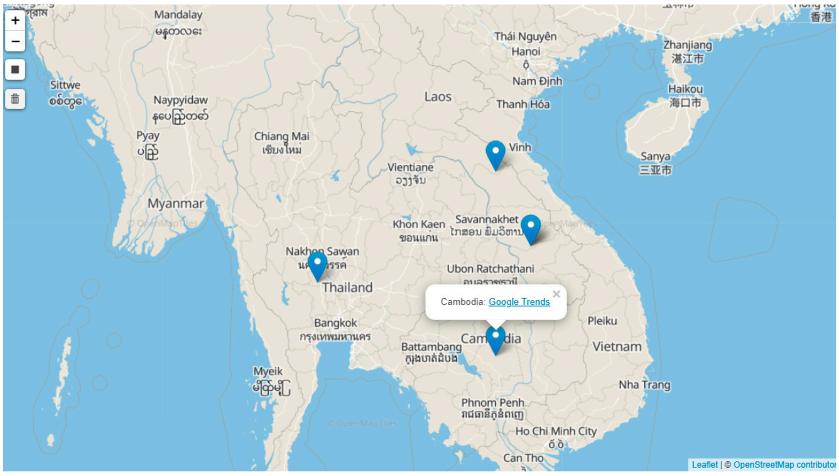

Before viewing the trends, we need to specify a time range in the **Time Selection** boxes (4). If we click on a blue marker, a popup will display the country name and present a link to retrieve/view data. If the link marked as Google Trends is clicked, a new window will open (popup should be allowed in the browser), which will show the trends of several search terms/keywords. Currently stored keywords are *dengue* and *fever*.

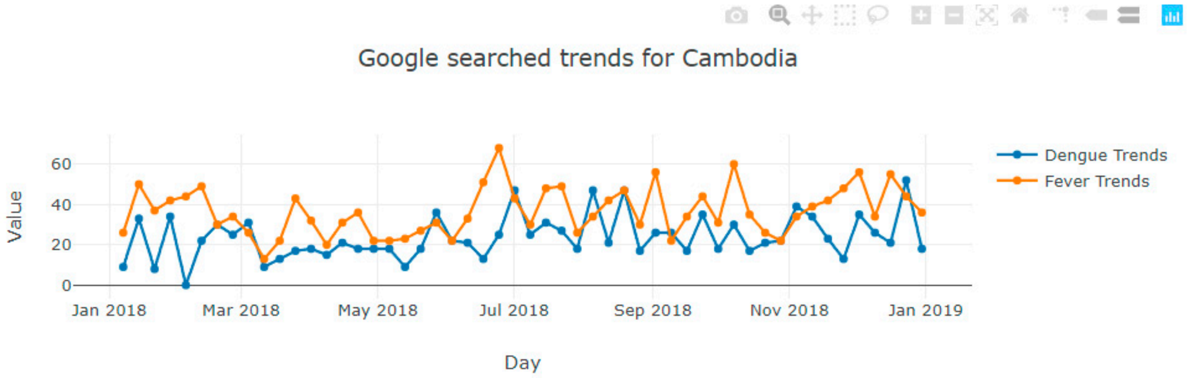

To understand how the trends are computed, please visit,

https://support.google.com/trends/answer/4365533?hl=en

Weather observations

Our server contains daily weather observation data on four key variables: average temperature, maximum temperature, minimum temperature, and precipitation. To view any of these data, first, we need to select a weather station. See the previous subsection on **Weather Stations** to understand the procedure of finding a station. Once a station is selected, the **Station Id** field in (6) will display the id. The id can also be manually entered.

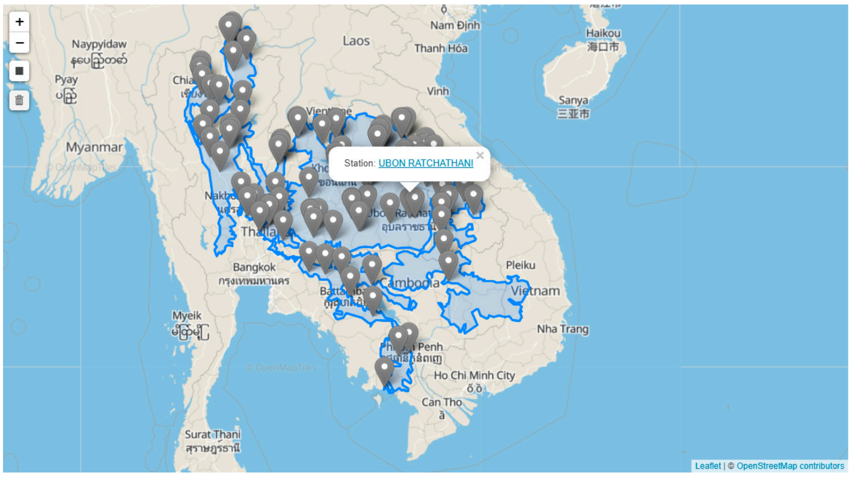

We also need to specify a time range in the **Time Selection** section in (4). Next, we need to select which weather variables we want to visualize from the **Ground Station Data** section in (6). We can choose one or any number of variables for visualization. Clicking the **Plot Data** button in (6) will send a request to the server with the selected parameters. If data are found, the PICTUREE--Aedes will open a new window (popup must be allowed in the browser) and show the requested plots.

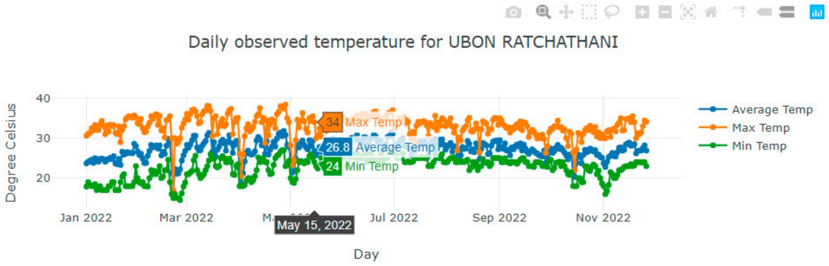

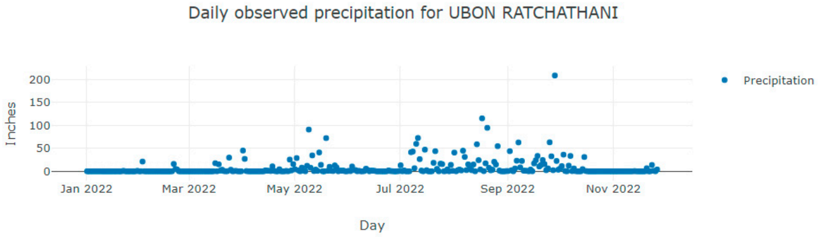

Reanalyzed data

There are four reanalyzed data available to be viewed with both spatial and temporal visualization tools. They are **Average Temperature**, **Dengue Reproduction Number**, **Aedes Mosquito Population**, and **Dengue Risk.**

For spatial visualization, a rectangular region must be marked in the map first. The upper left corner of the map window (1) contains a tool to draw and delete rectangles on the map. Using the 'Draw a rectangle' tool, a rectangular region can be drawn on the map using the mouse (left click mouse button and drag). Select a date (**Single Day**) from the **Time Selection** tool in toolbox area (4) and one reanalyzed data radio button (e.g. **Dengue Risk**) in UI region (7). Then, clicking **Plot Data** in the same section will show us the heatmap.

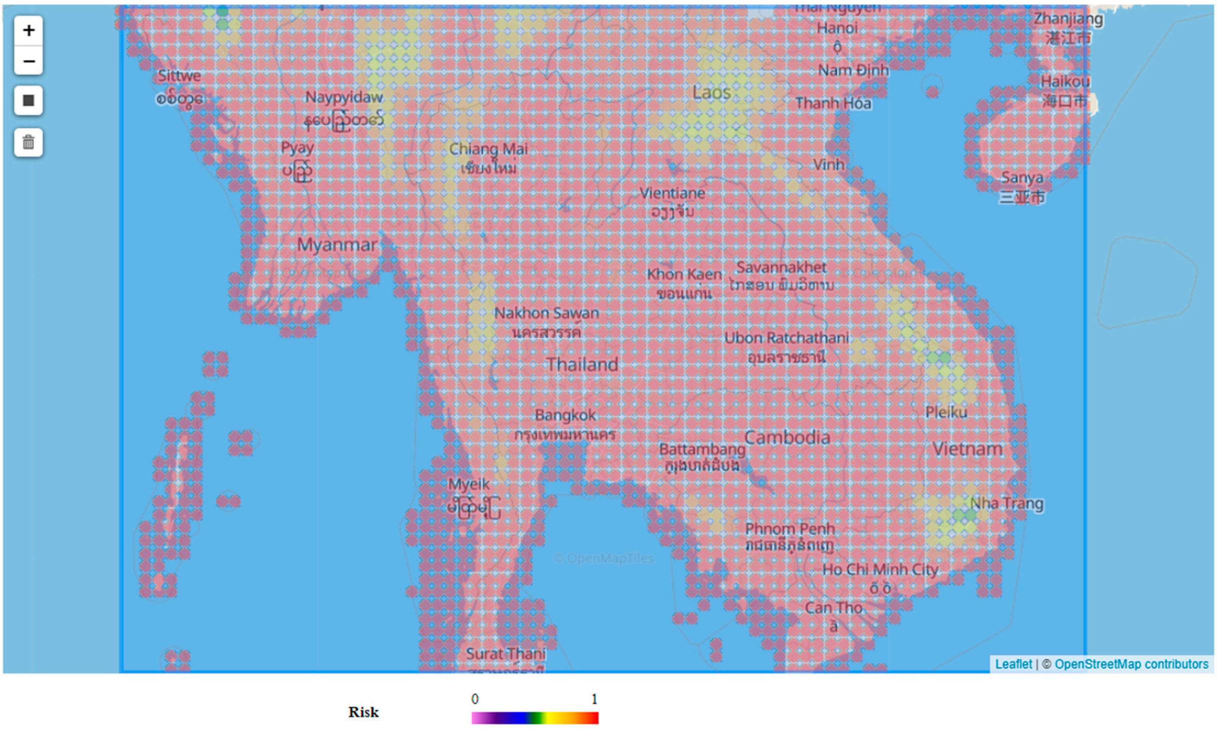

For temporal visualization, we need to select a time range from the **Time Selection** section tool (4). We also need to select a point by clicking anywhere on the map. The **Selected Point** section (3) will show the coordinates of the selected point. Next, we need to select one reanalyzed data radio button (e.g. **Dengue Risk**) from the **Time Series Analysis** section (8). Now, clicking **Plot Data** in the same section will show us the time series plot.

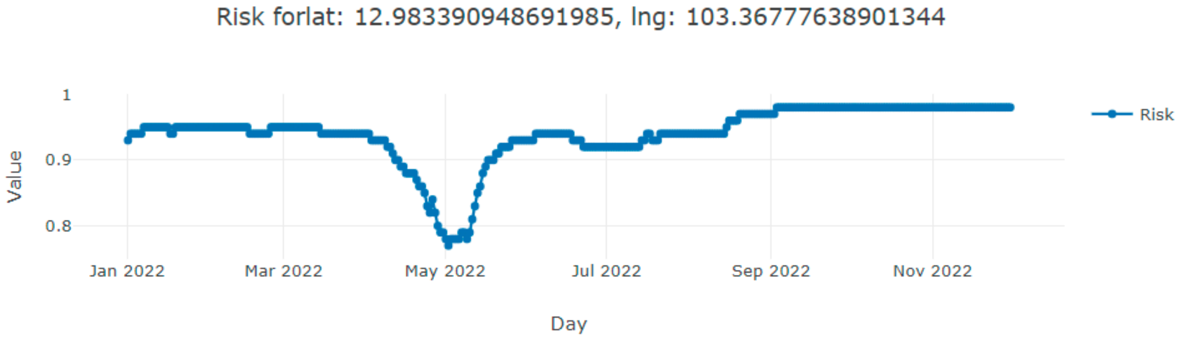

Steps to show other reanalyzed data are the same.

Vector map

The vector map data are collected from the Walter Reed Biosystematics Unit (WRBU). These maps provide global risk assessments of dengue vectors. We do not need a selection tool for these as the maps are loaded globally. First, we need to select a month and a vector species from the **Monthly Vectormap** section in (9).

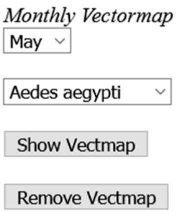

Clicking on the **Show Vectmap** button will load the mosquito vector (*Aedes aegypti*) map for the selected month on top of the global map.

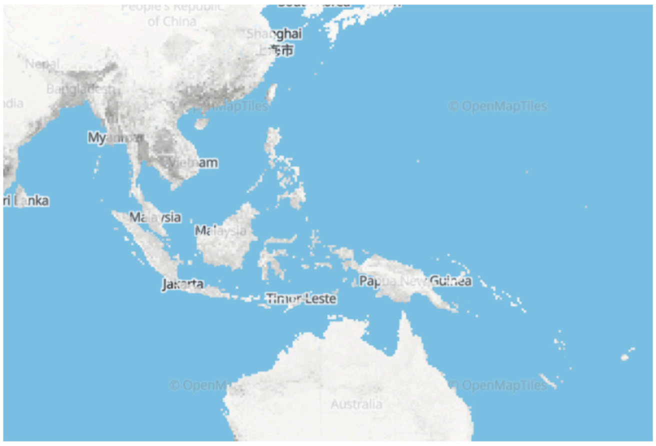

The darker shades indicate higher probabilities of vectors being present. Clicking the **Remove Vectmap** button will simply remove the overlay.

Dengue outbreak case prediction

The Dengue Predictions are designed to forecast the dengue incidence for ongoing dengue outbreaks. There are four application we built on different methods. They are ensemble Kalman filter, recurrent neural network, particle filter, and super ensemble. These predictions ask users to provide at least 10 reported cumulative case data, local population, last observation data, and clicking on the outbreak location on the map. The user can choose any prediction tool by click on the button. The results are the forecasted cumulative number of cases in the next few weeks. The forecasts are the distribution of cases and reports the lower quantile, median, upper quantile.

The EnKF prediction gives the distribution of the forecsts in the next 3 weeks with the quantiles and median.

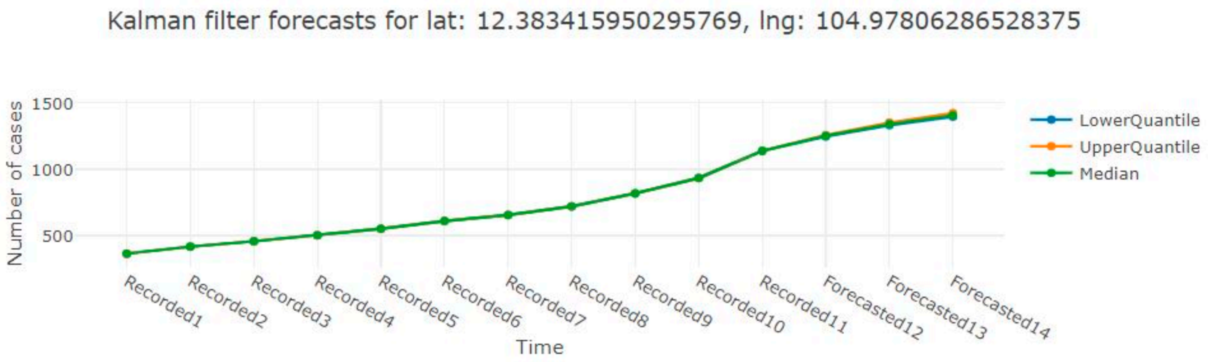

The neural network prediction gives the forecasts in the next 3 weeks.

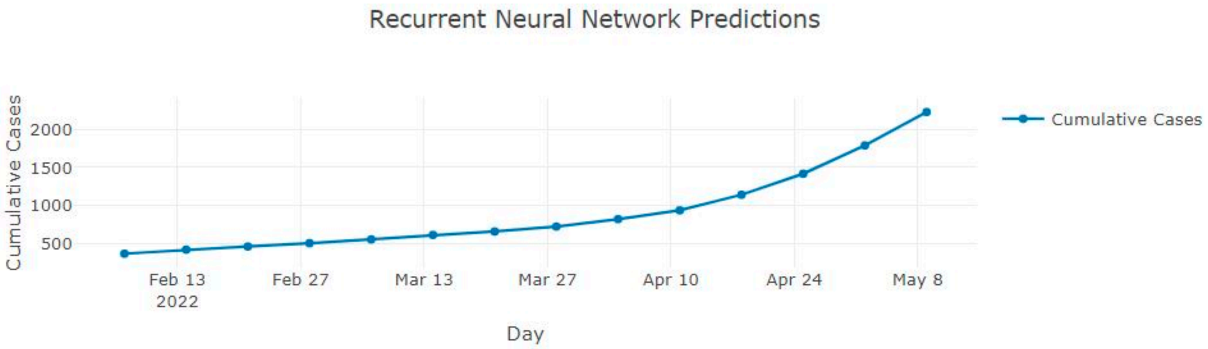

The particle filter prediction gives the distribution of the forecasts in the next 4 weeks.

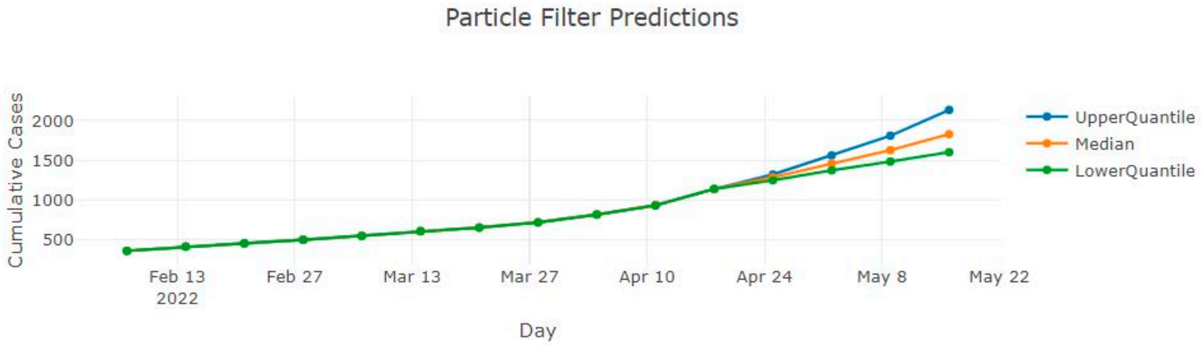

The super-ensemble prediction displays the median prediction from each member and the average and weighted average of the members

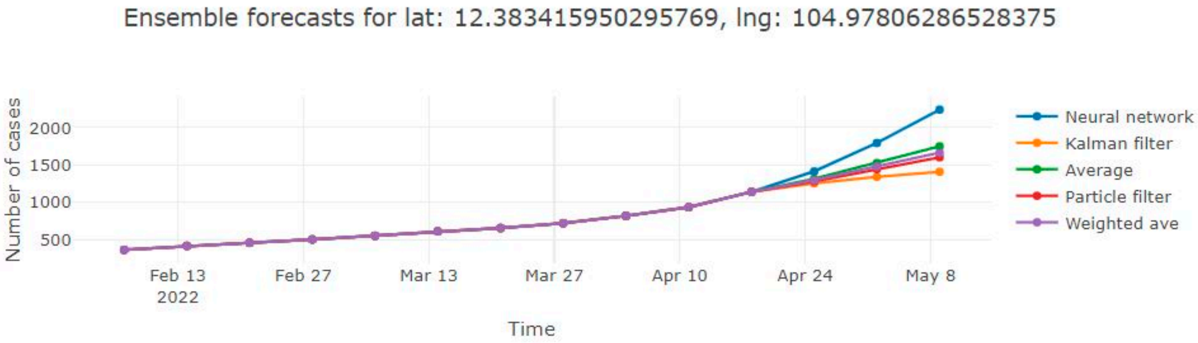

Dengue epidemic potential

The dengue epidemic potential (DEP) is the possibility with which dengue cases occur when competent mosquitoes (*Aedes*) are present. The simulation tool computes monthly DEP based on nearest weather station data. The DEP outcomes are defined as low, medium, and high. First, we must choose a limited selection tool: **Circle** or **Ecoregion** from (2). Next, we need to click on the map to designate a point. An area will be highlighted based on the selection tool. The UI area marked by (3) will show the coordinates of the point we clicked on. The UI area marked by (11) contains an input box to enter the search range where the system will automatically look for weather stations.

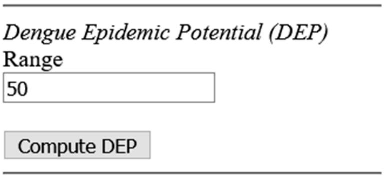

Now, if we click **Compute DEP** in the toolbox UI area (11), the PICTUREE--Aedes will send a request to the server to compute DEP for the designated point. When some results are available (which is typically within 5-10 seconds), a new window will open which will show the monthly DEP estimates.

Month	Dengue Epidemic Potential
Jan	Low
Feb	Medium
Mar	Low
Apr	Medium
May	High
Jun	High
Jul	High
Aug	High
Sep	High
Oct	High
Nov	Medium
Dec	Low

The PICTUREE--Aedes will also highlight the circle or the ecoregion which contains the point based on the DEP of the present (current) month. For the three DEP categories: low, medium, and high; the highlight colors will be green, yellow, and red, respectively.

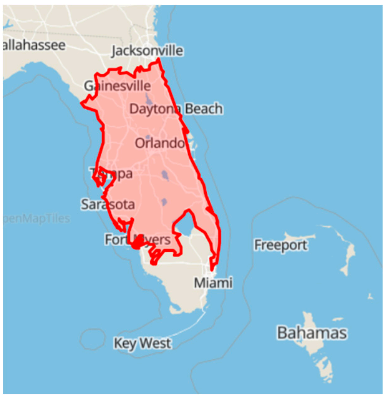

Supplement: Supplementary file 1 [file pathogens-12-00771-s001.zip › pathogens-2386411-supplementary.pdf]
